# Study on Meso-Material Parameters of Submarine Weathered Granite Based on Parallel Bond Model

**DOI:** 10.3390/ma15113878

**Published:** 2022-05-29

**Authors:** Shilei Zhang, Bonan Zhang, Bo Han, Qiyue Zhang, Di Liu

**Affiliations:** 1 Inner Mongolia Yinchuo Jiliao Water Supply Co., Ltd., Hinggan 137400, China; 201934648@mail.sdu.edu.cn; 2 School of Civil Engineering, Shandong University, Jinan 250061, China; 202020608@mail.sdu.edu.cn (B.Z.); 202135063@mail.sdu.edu.cn (Q.Z.); 202115014@mail.sdu.edu.cn (D.L.)

**Keywords:** offshore wind turbine, weathered granite, BPM, rock-socketed construction, uniaxial test

## Abstract

In order to study the mechanical properties of submarine weathered granite under marine geological conditions, uniaxial compression tests were carried out on the original medium weathered granite of the seafloor of an offshore area in Pingtan, Fujian Province by using triaxial experimental apparatus to analyze the fracture characteristics, stress–strain characteristics, and compressive strength indexes. Based on the theory of discontinuous medium, the uniaxial compression and uniaxial tensile tests of rocks were simulated, and the microscopic mechanical parameters of discrete elements of granite samples were determined based on the indoor macroscopic mechanical tests: effective modulus E_t_, compressive elastic modulus E_c_, macro Poisson’s ratio μ, and uniaxial compressive strength *σ*_c_. The results show that the parallel bond model has good simulation results for the uniaxial compression test, but the tensile strength and tensile–compression ratio were quite different from the experimental values. When the confining pressure is large, the calibrated parameter adaptability by uniaxial compression is poor. The reason for certain errors is a large resistance of the parallel bond model to particle rotation and the influence of normal stress on shear strength is not considered. The cementation model can be modified by adding coefficients based on laboratory test results.

## 1. Introduction

As a major renewable energy source, wind energy is considered the most effective way to reduce greenhouse gas emissions and has become an increasingly important renewable energy market in the past few decades [1].

The coastal wind resource in China (e.g., Fujian, Guangdong, and other coastal areas) is abundant and the offshore wind turbine (OWT) is being developed rapidly [2,3,4]. However, due to the influence of submarine islands and reefs, the topographic and geological conditions are complex [5,6], the rock formations are shallow, and the depth varies greatly. The properties of the overburdened soil are weak. Therefore, the rock-socketed construction method is needed for offshore wind turbine pile foundations [7].

However, the existing safety design and analysis methods of the offshore wind power pile foundation are more for soil seabed [8,9,10,11] and less for rock seabed. For the rock-soil mass of rock-based seabed, it is difficult to test due to the large difference in mechanical properties caused by different [12] weathering degrees. Additionally, under the extreme ocean load, the microscopic failure mechanism of weathered rock mass is not clear and the simulation accuracy is low. So, it is urgent to study the mechanical properties of rock-soil mass of rock-based seabed and clarify the mechanical properties and failure mechanism of submarine weathered granite. Rock macroscopic mechanical properties are affected to varying degrees by the differences in internal microscopic components properties of rock. Therefore, studying the fracture law of rock under various loads from the perspective of micromechanics is an important basis for revealing the fracture failure mechanism and constitutive behavior of rock, and it is also a key to establishing the relationship between macroscopic mechanical response and micromechanics of rock. In this paper, based on the original strongly weathered granite samples from offshore wind farms of an offshore area in Fujian Province, the mechanical models of uniaxial compression, triaxial compression, and Brazilian disc splitting of weathered rock masses were established by PFC and bonded-particle model to investigate the micromechanics.

## 2. Laboratory Tests

### 2.1. Physical Properties of Rock Samples

The test soil samples were taken from the engineering area of a wind farm in an offshore area in Pingtan Comprehensive Experimental Zone, Fujian Province where a large amount of late Yanshan granite is distributed. In the field, The rotary soil sampler with a single hole and double pipes was used, and the plant gum was supplemented as the circulating fluid for drilling and sampling several sections of weathered granite with depths of 30 to 35 m in the same borehole. The sampling method not only has a small disturbance on soil samples but also can ensure the undisturbed soil samples.

**Figure 1:**
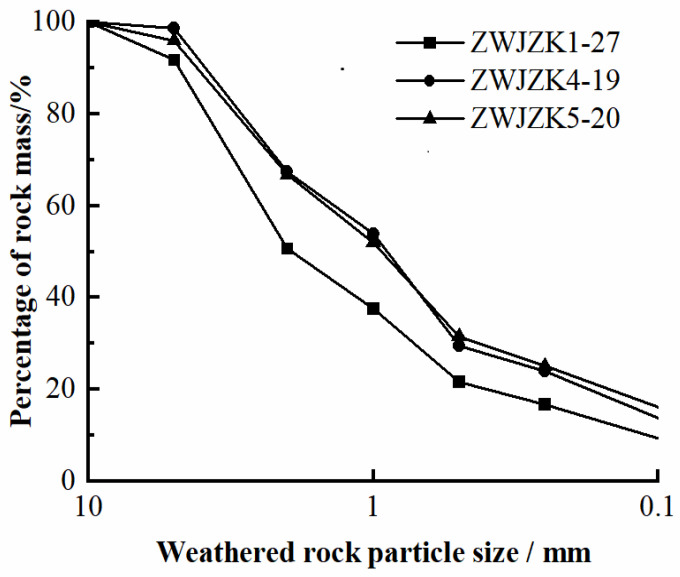
Analysis curve of weathered granite particles.

For the experiment, 27, 19, and 20 groups of samples (ZWJZK1-27, ZWJZK4-21, ZWJZK5-23) that contain residual soil, fully weathered soil, granular soil, and fragmented soil were taken from three different boreholes. The particle size distribution curve of weathered granite is shown in Figure 1. The percentage of rock mass larger than 1 mm in weathered rock particle size can reach 60%, and the percentage of rock mass smaller than 0.1 mm in weathered rock particle size is relatively small. The uneven coefficient C_u_ = 23 and the curvature coefficient C_c_ = 2.23 were obtained, which meet the requirements of good gradation.

### 2.2. Uniaxial Compression Test of Weathered Granite

**Figure 2:**
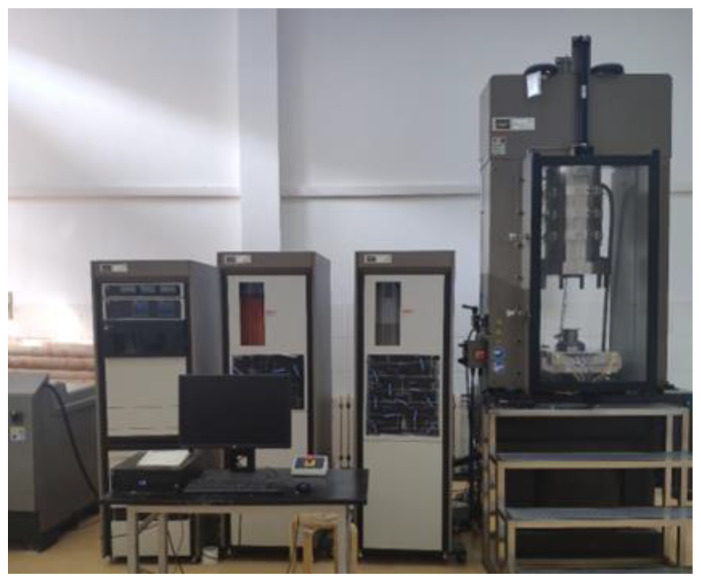
MTS815 all-digital hydraulic servo rock three-axis tester.

The whole process uniaxial stress–strain test was carried out by the MTS815 full digital hydraulic servo rock triaxial testing machine that consists of a loading system, testing system, control system, and program control system, independently developed by Shandong University, as shown in Figure 2.

The main parameters of MTS815 include: the maximum static axial test force is 2667 kN, the maximum dynamic axial test force is 1335 kN, the strain measurement range is ±0.03 mm, and the displacement measurement range is ±50 mm. The static test force accuracy is 1%, and the dynamic test force accuracy is 2%. The uniaxial stress–strain whole process test and triaxial stress–strain whole process test can be completed in MTS815. The programmable uniaxial and triaxial tests are carried out according to the requirements of the special test process. The axial load, axial displacement, the stroke of axial large range, and the circumferential displacement can be switched without impact in the control.

**Figure 3:**
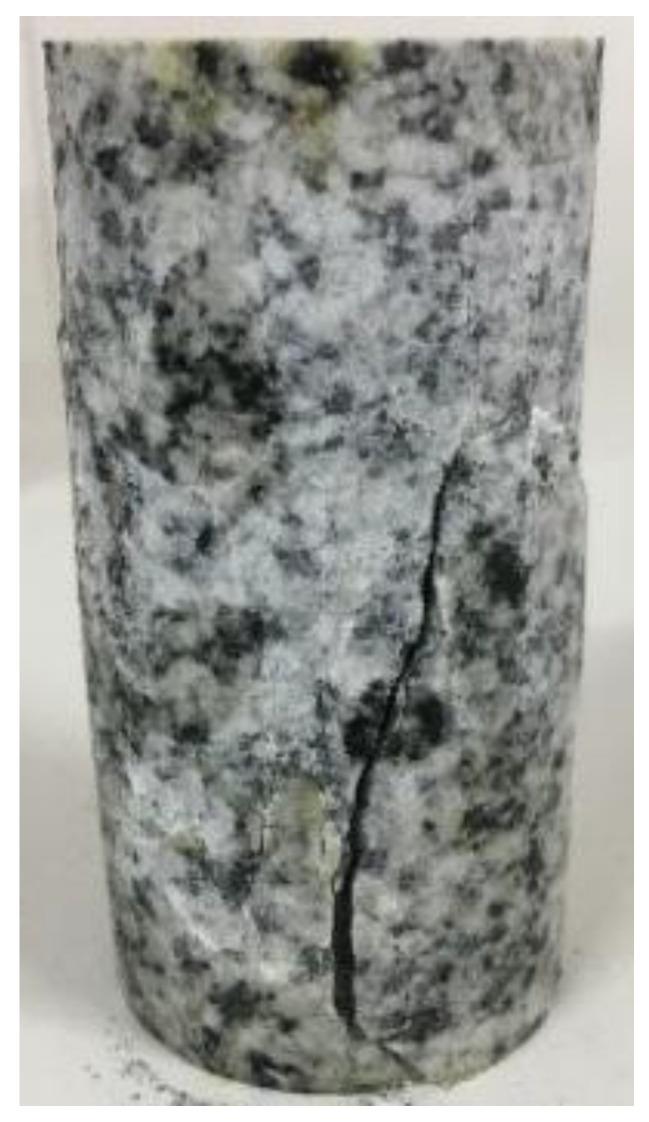
Uniaxial test results of weathered granite.

The height and diameter of weathered granite are 100 mm and 50 mm, respectively. The sample was packaged with rubber film and installed on the base for fixing. The sensor was installed, and the sensitivity of the sensor was tested from the control system. It was confirmed that the sensor could operate the test instrument for loading after normal operation. After setting the confining pressure in the working condition, at the beginning of the test, the confining pressure was applied to the set value at 3 MPa/min, and then the axial pressure was applied at 30 kN/min. When the specimen entered the yield stage (exceeding the peak value), the circumferential displacement rate was adjusted to 0.04 mm/min. When the specimen reached peak stress, in order to obtain the residual strength quickly, the axial displacement rate was adjusted to 0.08 mm/min until the specimen was damaged (Figure 3).

**Figure 4:**
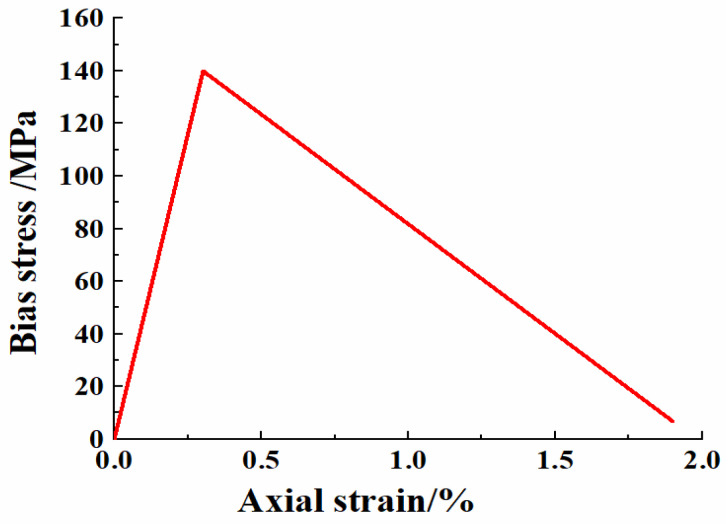
Uniaxial test stress–strain curve of weathered granite.

In the small-strain stage, the stress–strain of the medium weathered rocks in both groups in the test showed an obvious linear relationship (Figure 4), with the peak partial stress of 139.92 MPa, and the strains reaching the peak partial stress of 0.318% and 0.322%, respectively, which indicates stable rock properties. According to the stress–strain curve, the average deformation modulus and Poisson’s ratio of rock can be calculated to be 49.17 GPa and 0.266.

## 3. Numerical Model

The PFC program based on the discontinuous medium theory is one of the methods that can effectively analyze the macroscopic nonlinear mechanical behavior response of rock from the mesoscopic perspective. The analysis results are in good agreement with the actual results [13,14,15,16]. Therefore, the PFC program has been recognized and affirmed by researchers in the field of geotechnical engineering. In the detailed observation and study of rock mechanical properties using the PFC program, the selected model has an important influence on the accuracy of the final research results. At present, the commonly used models in the PFC program mainly include the traditional bonded particle model (BPM) [17,18], the flat-plate joint model (FJM), and the aggregation particle model (CPM). BPM has a low computational cost and does not require complex constitutive relations to describe the mechanical behavior of complex rock. Zhou [19,20,21] studied the fracture mechanism of prefabricated fractured rock mass under a series of different loads (uniaxial compression, Brazilian splitting, and triaxial compression) using traditional BPM. Park [22] introduced a rock structural plane based on the BPM model and conducted a large number of rock direct shear tests. To study the deformation and strength characteristics of granite under true triaxial loading, Zhang [23] performed 3D PFC numerical simulations using BPM. Zhang [24] discussed the acoustic emission characteristics of rocks under different compression loading rates based on BPM.

In this paper, the discrete element model of weathered granite was established by BPM model. Through the parameter calibration of the uniaxial compression test and uniaxial tensile test, the fitting formula of BPM model was obtained. Subsequently, Brazilian splitting and biaxial compression tests were simulated by the calibrated parameters, and the calculation results were analyzed according to the calculation principle of BPM model. Finally, the advantages, disadvantages, and reasons for the calculation results of BPM model were discussed, which provided reference and guidance for revealing the micromechanical properties of weathered granite.

### 3.1. Fundamentals of the Standard BPM

**Figure 5:**
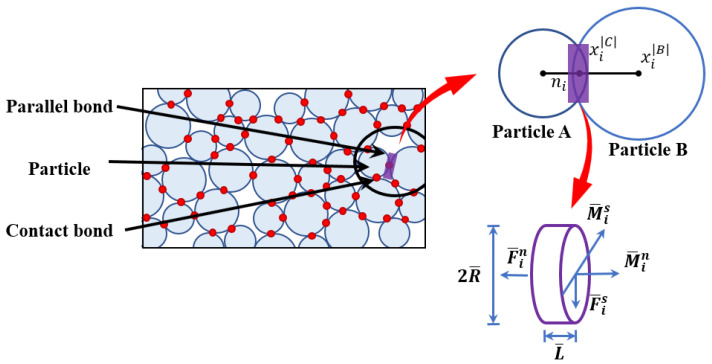
Force-displacement behavior of bonded-particle model. Modified after Potion and Cundall (2004) [13].

The parallel bond can be expressed as two sets of springs, one parallel to the contact surface between two contacting particles, and the other perpendicular to the surface. Each set of springs has its stiffness and strength parameters. When the stress acting on the bond exceeds the corresponding strength, the bond breaks. The maximum tensile and shear stresses (Figure 5) are calculated according to beam theory as follows.

(1) $$\sigma_{\max}=\frac{-\bar{F_{i}^{n}}}{A}+\frac{\left|\bar{M_{i}^{s}}\right|}{I}\bar{R}$$

(2) $$\tau_{\max}=\frac{\bar{F_{i}^{s}}}{A}+\frac{\bar{M_{i}^{n}}}{J}\bar{R}$$

where $\bar{F_{i}^{n}}$ and $\bar{F_{i}^{s}}$ are the normal and shear forces, respectively. The normal and shear forces of particle B; $\bar{M_{i}^{n}}$ and $\bar{M_{i}^{s}}$ are the bending and twisting moments acting at the center of the parallel bond; *A*, *I*, and *J* are the area of the bond cross section, the area of the moment of inertia and the polar inertia, the moment of inertia and the polar moment of inertia, respectively; and *R* is the average radius of particle A and particle B. If the maximum stress is greater than the corresponding strength $\sigma_{\max}>\bar{\sigma_{c}}$ or $\tau_{\max}>\bar{\tau_{c}}$, the parallel bond will fracture as a tensile or shear crack, respectively, and it will disappear or become a zero-length bond. At the same time, the bond stress will be zero and redistributed to the adjacent bond [25].

### 3.2. Calibration of Uniaxial Compression Test Parameters for Weathered Granite

**Figure 6:**
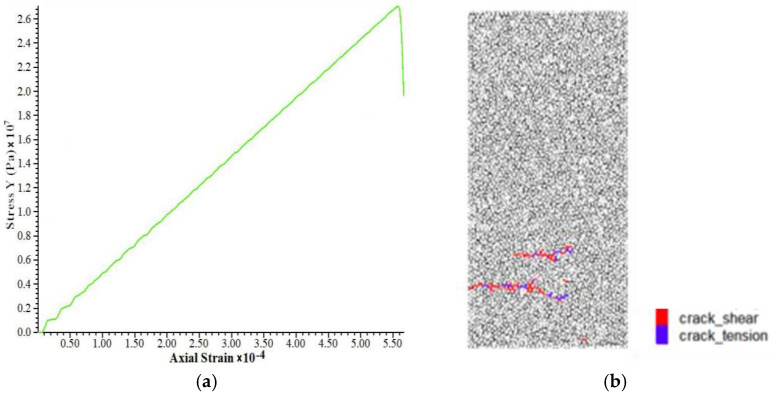
Calibration of uniaxial stretching parameters, (**a**) Stress strain curve, (**b**) break mode.

The two-dimensional model was used for calculations. According to the experimental data (Table 1), the specimen model was 50 mm wide and 100 mm high, and the particle radius was randomly distributed in the range of 0.5 to 5 mm. The void ratio was 0.296, and the density was 2280 kg/m^3^. According to the characteristics of the bonded-particle model, the tensile elastic modulus is first fitted by the stress–strain curve under direct tension. The linear stiffness (emod) was kept to a relatively small value (1 × 10^5^), and the parallel bonded effective modulus (Pb_deform) was adjusted to 1 × 10^9^, 5 × 10^9^, 10 × 10^9^, 20 × 10^9^, 40 × 10^9^, and 50 × 10^9^, and other parameters were taken to higher values. Stress–strain curves under direct tension were obtained (Figure 6).

**Figure 7:**
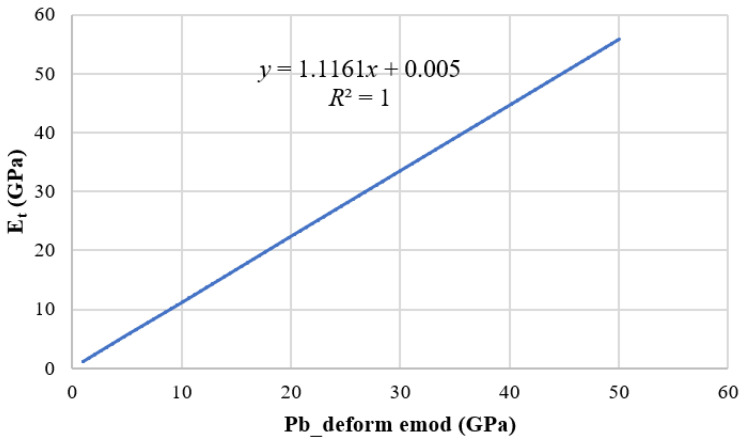
Correspondence between tensile modulus of elasticity and Pb_deform emo.

The green means shear crack and the blue means tension crack. The relationship between tensile elastic modulus and Pb_deform is obtained by fitting the peak stress and strain (Figure 7). It can be seen from the figure that the fitting formula is E_t_, E_t_ = 1.1161*x* + 0.005, where E_t_ is the effective modulus and the tensile elastic modulus to be calibrated is 49.17 GPa. Then, the tensile elastic modulus was substituted into the fitting formula, *x* should be approximately taken as 44.1 GPa, that is, the Pb_deform emod is 44.1 GPa. A new fitting formula is obtained by reducing the number of data sets. The uncertainty of a variable E_t_ with respect to parameter *x* is d = Δk/k = 10.12%, k is the fitting coefficient of *x.*

**Figure 8:**
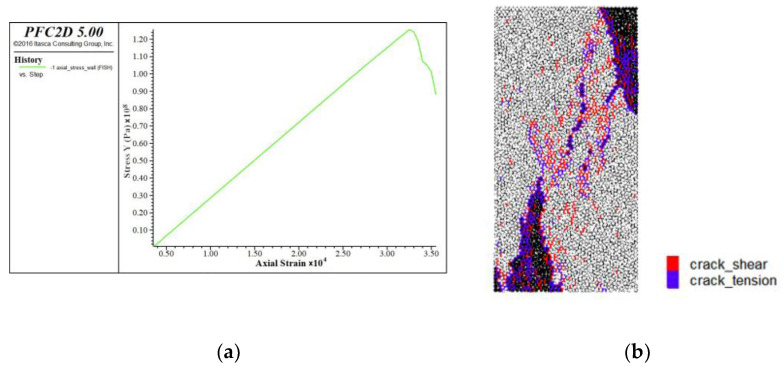
Calibration of Modulus of Elasticity in Uniaxial Compression, (**a**) stress strain curve, (**b**) break mode.

For the linear stiffness (emod) of the linear contact modulus, the size of the parallel bond modulus is kept unchanged (Pb_deform emod is 58.7 GPa), and the linear elastic modulus is changed to 1 × 10^9^, 10 × 10^9^, 20 × 10^9^, 40 × 10^9^ and 50 × 10^9^, respectively. The stress–strain curve and the failure curve of the specimen under uniaxial compression are obtained (Figure 8).

**Figure 9:**
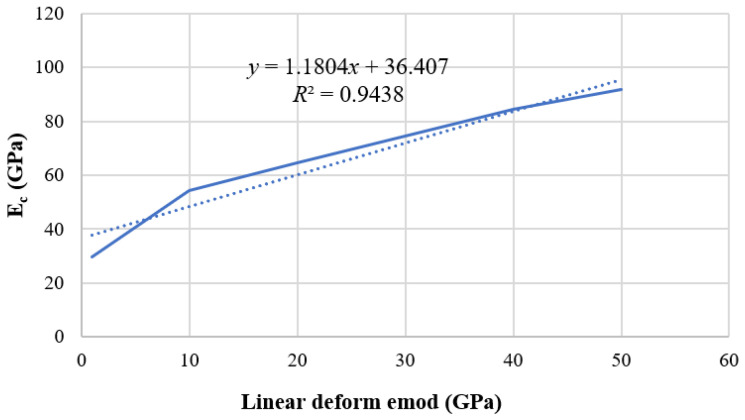
Relationship between compressive elastic modulus and linear contact modulus.

The red means shear crack and the purple means tension crack. The relationship between contact linear modulus and elastic modulus is shown in Figure 9. The dot line in the Figure 9 is the original data, and the solid line is the curve image after fitting. It can be seen from the figure that the fitting formula is E_c_ = 1.1804*x* + 36.407, where E_c_ is the compressive elastic modulus and *x* is the actual linear deform emod/1 × 10^9^. At this time, the tensile modulus to be calibrated is 49.17 GPa. Then, the tensile modulus was substituted into the fitting formula, *x* should be approximated as 10.8 GPa; that is, the linear deform emod is 10.8 GPa. A new fitting formula is obtained by reducing the number of data sets. The uncertainty of a variable E_c_ with respect to parameter *x* is d = Δk/k = 7.89%.

**Figure 10:**
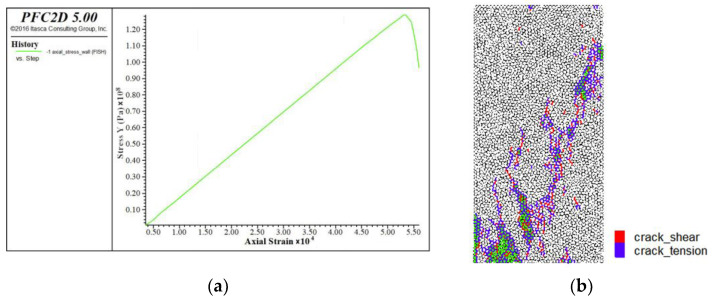
Uniaxial compression Poisson’s ratio parameter calibration, (**a**) curve figure, (**b**) failure mode.

For Poisson’s ratio, linear deform emod and Pb_deform emod were kept unchanged, assuming that the stiffness ratio (kratio) of parallel bond component and linear contact component were equal to study the corresponding relationship between stiffness ratio and macroscopic Poisson’s ratio. Setting stiffness ratios of 0.5, 1.0, 1.5, 2.0 and 3.0, uniaxial compression was carried out, and the failure results and stress curves of uniaxial compression are shown in Figure 10, where red means shear crack and purple means tension crack. In the figure, gray color represents particles, and blank color is pores.

**Figure 11:**
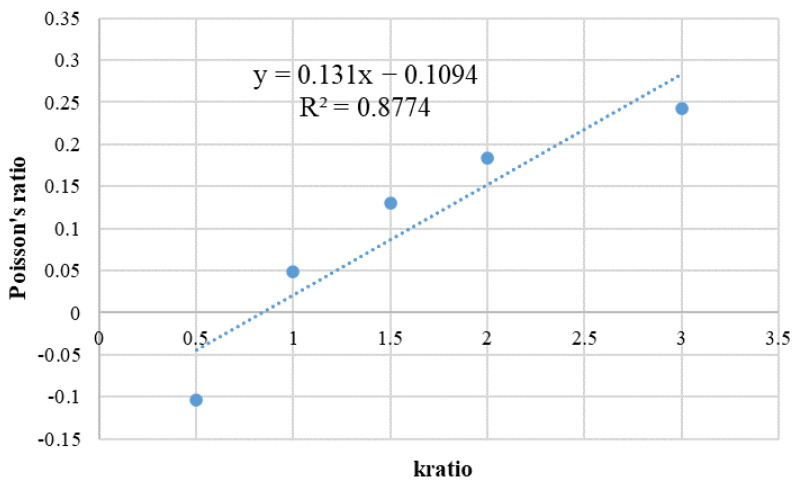
Correspondence between Poisson’s ratio and stiffness ratio.

Poisson’s ratio was calculated by taking the transverse and longitudinal nominal strains that correspond to half of the peak stress intensity, respectively, and the relationship between Poisson’s ratio and kratio is fitted as shown in Figure 11. The dot line in the Figure 11 are the original data, and the blue circles are the curve image after fitting From the figure, the formula is μ = 0.131*x* − 0.1094, where μ is macro Poisson’s ratio, and *x* is the stiffness ratio. The Poisson’s ratio value of 0.266 was substituted into the formula; the stiffness ratio can be obtained as 2.82. A new fitting formula is obtained by reducing the number of data sets. The uncertainty of a variable μ with respect to parameter *x* is d = Δk/k = 12.04%.

**Figure 12:**
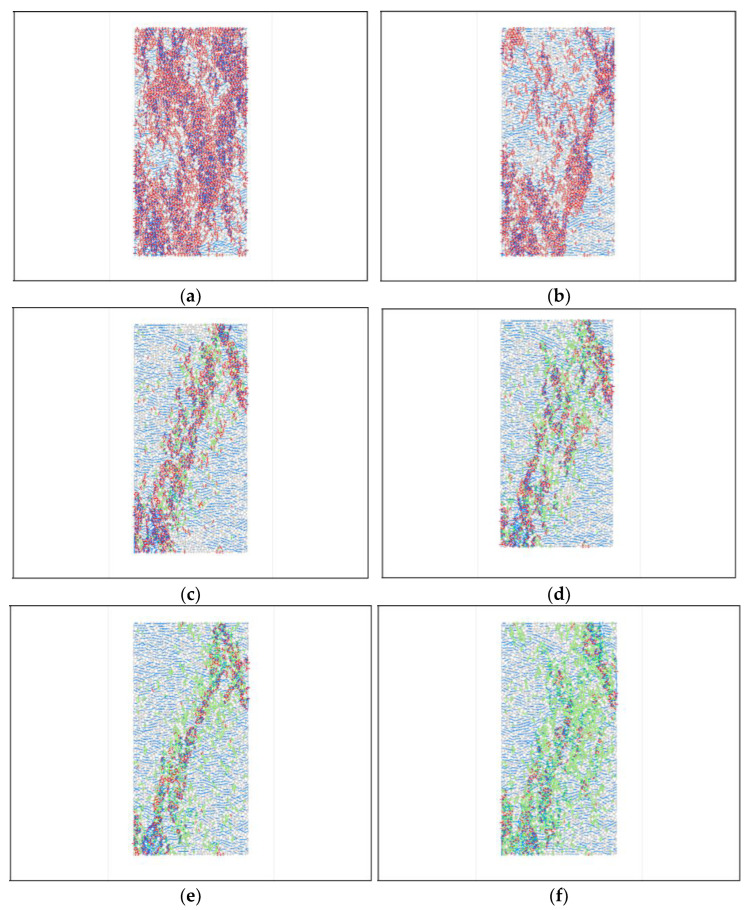
Parameter calibration of uniaxial compression bonding ratio, (**a**) ten_coh = 0.1, (**b**) ten_coh = 0.5, (**c**) ten_coh = 1.0, (**d**) ten_coh = 1.2, (**e**) ten_coh = 1.5, (**f**) ten_coh = 2.0. (As shown in the figure above, colored lines are cracks, and gray and white particles).

For the bonding ratio, after determining the effective contact modulus and stiffness ratio of linear bond and parallel bond, the normal bond or tangential bond strength is defined as the bonding ratio (ten_coh). To study the failure modes of specimens under different bond combinations, the bonding ratio was defined as 0.1, 0.5, 1.0, 1.2, 1.5, and 2.0, and the damage results of the specimens are shown in Figure 12, where green means shear crack and red means tension crack.

The results show that, when the bonding ratio is less than 1, the specimen is prone to tensile damage, and when the bonding ratio is larger, the specimen is prone to shear damage. According to the uniaxial compression damage of the rock in the actual specimen, the bonding ratio can be taken between 1.0 and 1.5, and the cohesion ratio is taken as 1.2 in this model.

**Figure 13:**
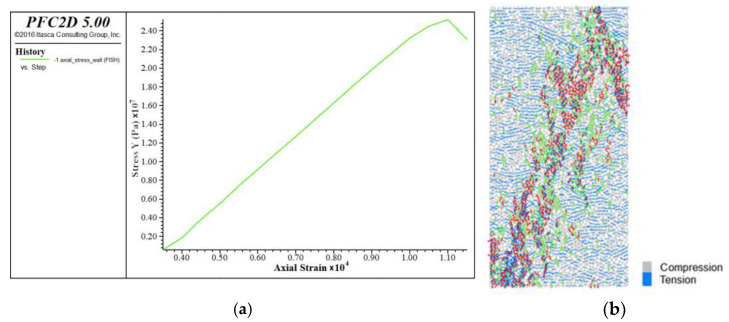
Parameter calibration of uniaxial compression magnification factor ratio. (**a**) stress strain curve, (**b**) break mode.

**Figure 14:**
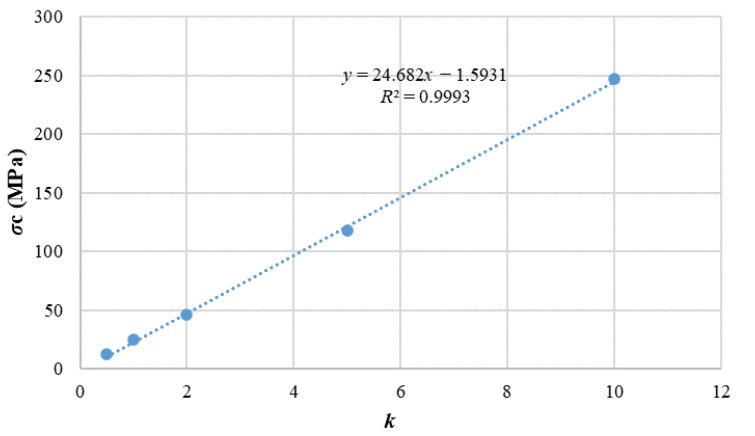
Fitting relationship between uniaxial compression magnification factor ratio and compressive strength.

For the amplification factor of bonding strength, the bonding ratio is fixed, assuming that the tangential bonding strength pb_coh = 1 × 10^7^ Pa, the normal bonding strength pb_ten = 1.2 × 10^7^ Pa can be obtained according to the bonding strength ratio 1.2, which will be positioned as the base bonding strength. On the basis of the reference bond strength, the normal bond strength and the tangential bond strength were multiplied by the magnification factor k, which was set to 0.5, 1.0, 2.0, 5.0, and 10.0, in turn. The failure modes and stress curves of different samples were obtained (Figure 13), where red means shear crack and green means crack-mix. In the figure, gray color represents particles, and blank color is pores.. The fitting relationship between the peak stress strength and the magnification factor is shown in Figure 14. The dot line in the Figure 14 are the original data, and the blue circles are the curve image after fitting. The fitting function *σ*_c_ = 24.682*x* − 1.5931 can be obtained, where *σ*_c_ is the uniaxial compressive strength of the rock and *x* is the cohesive strength ratio. The *σ*_c_ that value is 139.92 was substituted into the function, the *x* can be obtained as 5.74, the normal cohesive strength is 68.8 MPa, and the tangential cohesive strength is 57.4 MPa. A new fitting formula is obtained by reducing the number of data sets. The uncertainty of a variable *σ*_c_ with respect to parameter *x* is d = Δk/k = 9.61%.

**Figure 15:**
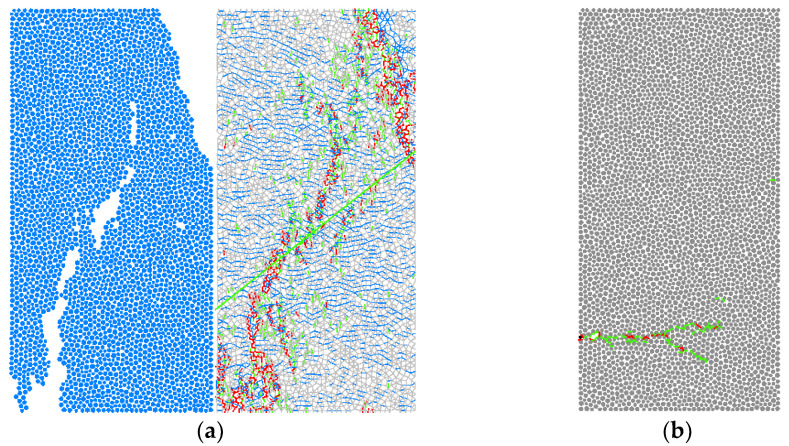
Comparison of rupture in uniaxial test and simulation results, (**a**) single-axis compression simulation, (**b**) uniaxial tensile simulation. (As shown in the figure above, colored lines are cracks, and gray and white particles).

After the preliminary calibration, the uniaxial compression test and uniaxial tensile test (Figure 15) were carried out by using a series of mesoscopic deformation and bonding parameters determined previously.

For the uniaxial compression fracture of the specimen, the simulation results are basically consistent with the experimental results (Figure 3) that the specimen presents one main crack. As can be seen from the simulation results, the cracking type is a tensile–shear composite failure, which is consistent with the research results of Einstein [26]. The elastic modulus under compression was 47.01 GPa, Poisson’s ratio 0.241, compressive strength 136.5 MPa, and tensile elastic modulus 42.94 GPa. Since each parameter will also interact with each other, the compressive elastic modulus and tensile elastic modulus obtained by the combination were slightly smaller than those under 65.5 GPa, and the compressive strength was slightly smaller than that under 185 MPa.

## 4. Simulation of Biaxial Compression Test

**Figure 16:**
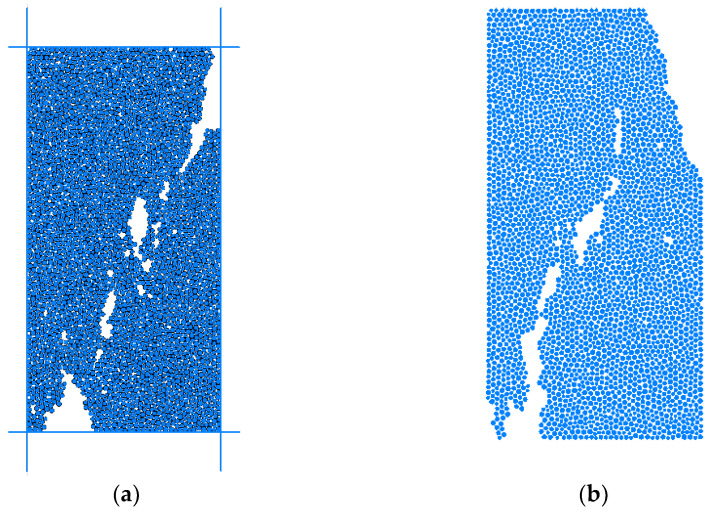
Comparison of rupture in uniaxial test and biaxial results, (**a**) biaxial compression test, (**b**) Single-axis compression results.

The biaxial test parameters are shown in Table 2. Based on the above parameters, the biaxial test was carried out. Firstly, the confining pressure is set to be small (1 × 10^6^), which was compared with the uniaxial test results as shown in Figure 16.

**Figure 17:**
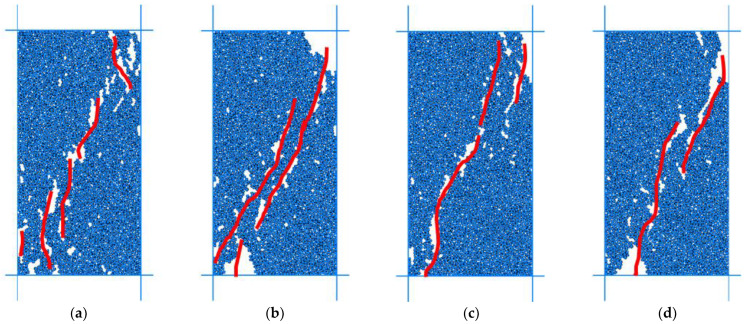
Comparison of biaxial test results under different confining pressures, (**a**) 1 × 10^6^ Simulation results, (**b**) 10 × 10^6^ simulation results, (**c**) 20 × 10^6^ simulation results, (**d**) 30 × 10^6^ simulation results. (As shown in the figure above, colored lines are cracks, and gray and white particles).

**Figure 18:**
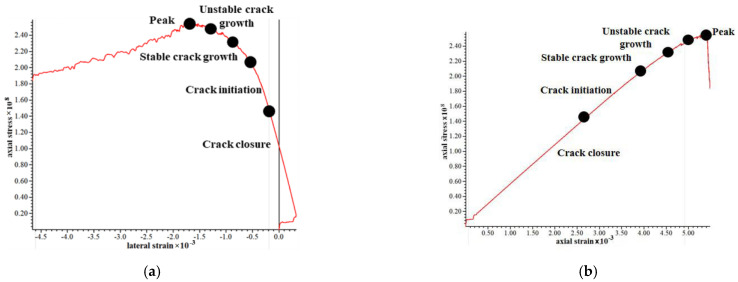
Stress–strain curve at 20 × 10^6^ confining pressure. (**a**) horizontal stress-strain curve, (**b**) vertical stress-strain.

It can be concluded that the compressive strength is 132.37 MPa, and the simulation results are slightly larger than the uniaxial test by 4%. According to the elastic modulus and confining pressure under various working conditions, the fracture results are shown in Figure 17. The stress–strain curve under confining pressure of 20 × 10^6^ was shown in Figure 18 and the calculation results were shown in Table 3.

In the biaxial test, the error of compressive strength increases with the increase in confining pressure using the parameters calibrated by uniaxial compressive strength, and the compressive strength also increases with the increase in the internal friction angle. When the confining pressure is 30 × 10^6^, the compressive strength is 302.7 MPa, and the error between the results and the experimental value is 29.5%. When the friction angle increases from 50.6 to 70.6, the compressive strength increases to 340.1 MPa, and the error between the results and the experimental value is 21%, there is still a large error. If the friction angle continues to increase, the compressive strength increases slightly. The main reason for this phenomenon is that the BPM model uses a finite length bond to simulate the contact behavior between the particles, the effect of which is equivalent to two sets of springs, as shown in Figure 5. One set of springs is parallel to the contact surface of the particles, and the other is perpendicular to the contact surface, with each set of springs being the carrier of the force applied so that it can simultaneously withstand the clutch torque. However, the slip model cannot exist at the same time as the parallel bond model, and the slip model only works after the parallel bond failure. The BPM model calculates the maximum tensile stress and shear stress according to the beam theory. In this process, bending moment and torque contribute the most to the maximum tensile stress and shear stress, respectively. The parallel bond will cause the excessive rotation resistance of spherical particles (spherical particles will produce excessive rolling after the fracture of the parallel bond), and the shear strength of the BPM model depends on the bond strength of parallel keys, which is a constant, because the shear strength is not related to the normal stress, and the friction strength only plays a role after the parallel bond failure, which is different from the actual mechanical behavior. These all lead to some differences between the calculated results of BPM model and the actual results in the tension–compression ratio and biaxial calculation. However, for the uniaxial compression test, the simulation results are in good agreement with the experimental results, which can reveal the meso-failure mechanism.

## 5. Conclusions

1. The parallel bonding model was calibrated by uniaxial testing, which found the elastic modulus, Poisson’s ratio, and compressive strength were in good agreement with the experimental values. However, the tensile strength and tensile-compressive ratio differed significantly from the experimental values, and fine-tuning was still unable to achieve an error within 10%.
2. To simplify the experimental process, The parameters obtained by uniaxial compression were used for biaxial tests. By changing the confining pressure, the calculated compressive strength is compared with the results of uniaxial compression. When the enclosing pressure was very small, the calculated compressive strength was closer to the strength of uniaxial compression. However, when the enclosing pressure increased, the simulation results are quite different from the experimental results and are smaller than the experimental values. By increasing the friction angle, the calculated compressive strength increased, but the error was still large.
3. The reason for a certain gap between the calculation results and the experimental results is the large resistance of the parallel bonding model to particle rotation, and to consider the effect of normal stress on shear strength. The bonding model can be modified by adding coefficients based on laboratory test results.

## Figures and Tables

**Table 1:** Basic physical properties of weathered granite.

Density *ρ*/(g/cm^3^)	Specific Gravity *G*_s_	Water Content *ω/*%	Porosity Ratio *e*	Liquid Limit *ω*_L_/%	Plastic Limit *ω*_P_/%
2.28	2.68	9.7	0.296	32.7	20.2

**Table 2:** Macro parameters of biaxial compression test.

Sample No.	Confining Pressure MPa	Compressive Strength MPa	Elastic Modulus GPa	Cohesion MPa	Friction Angle °
2-1	10	253.34	48.31	28.13	50.6
2-2	20	307.57	51.05		
2-3	30	429.52	58.54		

**Table 3:** Calculation results of compressive strength of biaxial test.

Surrounding Pressure	*σ*_c_ Test Value/MPa	*σ*_c_ Simulated Value/MPa	Error
1 × 10^6^	132.37	143	4%
10 × 10^6^	253.34	207.7	18%
20 × 10^6^	307.57	258.9	15.8%
30 × 10^6^	429.52	302.7	29.5%

## Data Availability

Not applicable.

## Nomenclature

*ρ*	Density
*G*_s_	Specific gravity
*ω*	Water content
*e*	Porosity ratio
*ω*_L_	Liquid limit
*ω*_P_	Plastic limit
C_u_	Uneven coefficient
C_c_	Curvature coefficient
$\bar{F_{i}^{n}}$	Normal force
$\bar{F_{i}^{s}}$	Shear force
$\bar{M_{i}^{n}}$	Bending moment acting at the center of the parallel bond
$\bar{M_{i}^{s}}$	Twisting moment acting at the center of the parallel bond
E_t_	Effective modulus
E_c_	Compressive elastic modulus
μ	Macro Poisson’s ratio
*σ*_c_	Uniaxial compressive strength
